# Depression, Anxiety, Stress among Postgraduate Medical Residents: A Cross Sectional Observation in Bangladesh

**Published:** 2019-07

**Authors:** Mohammad Shibli Sadiq, Nahid Mahjabin Morshed, Wasima Rahman, Nafia Farzana Chowdhury, SMY Arafat, Mohammad S.I. Mullick

**Affiliations:** 1 National Institute of Mental Health, Dhaka, Bangladesh.; 2 Department of Psychiatry, Bangabandhu Sheikh Mujib Medical University, Dhaka, Bangladesh.

**Keywords:** *Anxiety*, *Bangladesh*, *Depression*, *Residents*, *Stress*

## Abstract

**Objective:** Medical training has been reported as being stressful, and postgraduate medical training environment has always been regarded as highly stressful, which may lead to different psychiatric disorders. In this study, it was aimed to determine the extent of depression, anxiety, and stress among the postgraduate medical residents of Bangladesh.

**Method** **:** This cross sectional study was conducted at the Department of Psychiatry of Bangabandhu Sheikh Mujib Medical University, Dhaka, Bangladesh. Data were collected from 200 residents through face to face interview which was conducted by a psychiatrist using research instruments. Initially, respondents were approached by cluster sampling. Then, they were selected from each cluster using simple random sampling method (lottery method). Sample size was calculated by considering the prevalence of 50%; however, 200 residents were interviewed due to time constraints. The questionnaire consisted of 3 parts: (1) demographic variable, (2) the structured clinical interview for DSM-IV Axis-I disorders, and (3) Bangla Depression Anxiety Stress Scale (DASS-21). Data were analyzed using software Statistical Package for Social Sciences version 16.0.

**Results: **Distribution of depression, anxiety, and stress revealed that 11.5% of the residents had depressive disorders, 11% anxiety disorders, and 10.5% stress disorders. Also, it was found that 14.5% of the respondents were found to have at least one of three disorders. The DASS-21 score revealed that 6% of the residents had severe to extremely severe depression, 3.5% severe to extremely severe anxiety, and 6.5% severe to extremely severe stress disorder.

**Conclusion: **The study revealed that one in every seven residents has been suffering from at least one disorder from depression, anxiety, and stress related disorders. A large-scale multicenter study is recommended to validate the findings of the present study.

Medical training has reportedly been found stressful, which may be further increased during residency training as a result of increased expectations and responsibilities (1, 2). Residents are expected to be proficient clinicians, educators, researchers, and administrators at the end of their training (1). Postgraduate medical training environment has always been regarded as highly stressful to students. The consequences of high level of perceived stress include depression, burn-out, anger/irritability, anxiety, poor sleep, fatigue, and substance abuse (3, 4). Sleep deprivation has also been shown to predispose residents to medical errors, injuries, increased alcohol, and drug use, and increased conflict with other health care staff (5).

Studies show that doctors who work with reduced levels of mental concentration can be harmful to themselves, their colleagues, and patients (6). Although the actual incidence of depression in physicians is unknown, several studies demonstrated that one-fourth to one-third of residents may be clinically depressed at some point of their training (6). Studies conducted in Western countries on trainees in family practice, internal medicine, pediatrics, dentistry, emergency medicine, and surgery residencies have demonstrated that 3% to 35% have been suffering from significant depression and/or anxiety during their training, which can even lead to suicide (7).

In a meta-analysis of 25 studies from 1960 to 2003, rates of male physician suicide was 1.4 times higher than the average population and 2.3 times higher than female physicians (8).

Bangabandhu Sheikh Mujib Medical University (BSMMU), a 1500-bedded tertiary level hospital and the only medical university in Bangladesh, started its postgraduate residency program in March, 2010 aiming at preparing proficient and skilled doctors who can provide care to patients and ensure the health of the society as a whole. At this time, the university has 10 faculties with 59 specialties. Since 2010, every year, approximately 150 residents take admission in different disciplines of Medicine and Surgery Faculty of BSMMU. There are 19 disciplines in Faculty of Medicine and 12 in Faculty of Surgery. This residency system of medical education is competency-based and outcome-oriented, and it is expected to provide good quality health care to its consumers. However, studies conducted around the world on psychiatric disorders suggest that the current training may have an unintentional negative impact on trainees’ mental health, predisposing them to depression, anxiety, and stress (9). As the residency program is relatively new in Bangladesh, no formal study has yet been conducted to examine psychiatric disorders among residents. Thus, the present study was designed to determine the extent of depression, anxiety, and stress among the residents of BSMMU. 

## Materials and Methods

***Ethical Aspects***

Researchers were concerned about the ethical aspects of the study. The study was approved by the Institutional Review Board (IRB) of BSMMU (BSMMU/2014/246). Informed written consent was obtained from the participating residents and data were collected anonymously. Confidentiality of data was ensured and unauthorized access to data was not allowed.

***Study Population and Data Collection***

This cross sectional study was conducted from August 2012 to September 2014 in Psychiatry Department of BSMMU, Dhaka, Bangladesh. There are 19 disciplines in Faculty of Medicine and 12 in Faculty of Surgery. Since 2010, every year, approximately 250 residents take admission in different disciplines of Faculties of Medicine and Surgery. Sample size was calculated by considering the prevalence of 50%; however, 200 residents, instead of 384, were interviewed due to time constraints. Data were collected from 200 residents: 100 from Phase A and 100 from Phase B through face to face interview and using research instruments to collect data on variables of interest. Residents from Faculties of Medicine and Surgery were recruited. Firstly, the broad group was considered as Phase A and Phase B residents. Then, the residents of both phases were clustered based on their department. The required number of residents were then selected from each cluster using simple random sampling method (lottery method). Data were analyzed using software Statistical Package for the Social Sciences (SPSS) version 16.0. 

***Research Instruments***

- A semi-structured questionnaire to evaluate the sociodemographic variables.
- SCID-I was used by the researcher for the diagnosis of DSM-IV Axis-I disorders.
- Depression Anxiety Stress Scale (DASS-21) Bangla was filled by the respondents. DASS-21 (English version) was used for foreign residents (10, 11).

**A.  Semi-structured Questionnaire for Sociodemographic variables** **:**

The questionnaire had sociodemographic variables such as age, gender, nationality, residence (rural or urban), religion, marital status, monthly salary/income, status of the parents (alive/ died/ separated), family history of mental illness, and recreational facilities. 

**B.  SCID- I: The Structured Clinical Interview for DSM-IV Axis-I Disorders:**

The structured clinical interview for DSM-IV Axis-I disorders is a semi-structured interview for making major DSM-IV Axis-1 diagnosis. SCID is available in 2 versions: clinical and research. The Research version is divided into 6 modules, Module A: mood episodes; Module B: psychotic symptoms; Module C: psychotic disorders; Module D: mood disorders; Module E: substance use disorders; Module F: anxiety and other disorders. In this study, the first author who is a psychiatrist used Modules A, D, F of research version of SCID-I. 

**C.  DASS-21 Bangla Version** **:**

The short-form of DASS-21 was developed in English by Prof. H Lovibond & Prof. P. F. Lovibond at the University of New South Wales Australia. This version of DASS is a valid 1 set of 3 self-report scales with 21 items, which was designed to measure the negative emotional states of depression, anxiety, and stress (10). Each of the items rates, on a 4-point Likert scale, the frequency or severity of the participants' experiences over the last week with the intention of emphasizing states over traits. These scores ranged from 0, meaning that the client believed the item did not apply to him/her at all to 3 meaning that the client considered the item to apply to him/her very much or most of the time", while items 1 and 2 fell somewhere in between. Also, in the instructions it is stressed that there is no correct or wrong answer. There are 7 questions in each subscale. The sum of the scores for each of the 7 questions completed by each participant in each of the Sub-4-Scales are evaluated as per severity rating index below. The Bangla DASS-21 was validated by Alim et al, in 2014 (11).

## Results

In the present study, a total of 256 residents were approached from the sampled population. Among them, 200 participated in the study; the response rate was 78%. Among the residents, 11.5 had depressive disorders, 11% anxiety disorders, and 10.5% stress disorders based on the SCID-I diagnoses (Table 2). However, the point prevalence of the three disorders was found to be 14.5%. Major depressive disorder (MDD) single episode, MDD recurrent episode, and depressive disorder not otherwise specified (MDD NOS) were found in 3.5% of cases. Under the heading of anxiety disorders, 8.5% had specific phobia and 2.5% generalized anxiety disorder (current only), and under the heading of stress disorders, 7.5% residents had adjustment disorder (current only) (Table 2). According to DASS-21 score, 2% of the residents had mild, 3.5% moderate, 3% severe, and another 3% had extremely severe depressive disorder. Moreover, 1.5% had mild, 5.5% moderate, 2% severe, and another 1.5% had extremely severe anxiety disorder. Also, of the residents, 2% had mild, 2.5% moderate, 6% severe, and 0.5% had extremely severe stress disorder (Table 3). 

**Table 1:** Demographic Characteristics of the Postgraduate Medical Residents in Bangladesh (n = 200)

**Sociodemographic** **characteristics**	**Frequency**	**Percentage**	**Mean ± SD**
**Age (years)** ^*^			
< 25	5	2.5	
25 – 30	65	32.5	
30 – 35	74	37.0	32.6 ± 4.1
35 – 40	46	23.0	
≥ 40	10	5.0	
**Sex**			
Male	150	75.0	
Female	50	25.0	
**Nationality**			
Bangladeshi	178	89.0	
Other nationalities	22	11.0	
**Faculty**			
Medicine	140	70.0	
Surgery	60	30.0	
**Resident**			
Hostel	52	26.0	
Family	138	69.0	
Alone	10	5.0	
**Religion**			
Islam	161	80.5	
Hindu	35	17.5	
Others	1	0.5	
**Marital status**			
Married	169	84.5	
Unmarried	30	15.0	
Divorced	1	0.5	
10000	45	22.5	
10001 – 25000	74	37.0	
>25000	59	29.5	
No income	22	11.0	
NA	22	11.0	

**Table 2:** Depressive, Anxiety, and Stress Disorders among Postgraduate Medical Residents in Bangladesh as Per SCID-1 Diagnoses

**Psychiatric disorder pattern**	**Frequency**	**Percentage**	
**Disorder**			
Depressive disorders	23	11.5	
Anxiety disorders	22	11.0	
Stress disorders	21	10.5	
Overall disorder	31	14.5	
**Number of disorders**			
Single disorder	8	4	
Double disorders	11	5.5	
Multiple disorders	12	6	
**Depressive disorders**			
Major depressive disorder (MDD), single episode	7	3.5	
MDD, recurrent episode	7	3.5	
DD NOS	7	3.5	
**Anxiety disorders**		
Panic disorder	3	1.5
Social phobia	5	2.5
Specific phobia	17	8.5
Obsessive Compulsive Disorder	3	1.5
Generalized anxiety disorder	5	2.5
Anxiety disorder due to General Medical Condition	1	0.5
**Stress disorders**		
Posttraumatic stress disorder	2	1.0
Adjustment disorder (current only)	15	7.5

**Table 3:** Severity of Depression Anxiety and Stress According to DASS-21 among Postgraduate Medical Residents in Bangladesh

**DAS 21 score (Depression, Anxiety & Stress)**	**Frequency**	**Percentage**
**DASS 21 score (Depression)**		
Mild (5-6)	4	2.0
Moderate (7-10)	7	3.5
Severe (11-13)	6	3.0
Extremely severe (≥14)	6	3.0
**DASS 21 score (Anxiety)**		
Mild (4-5)	3	1.5
Moderate (6-7)	11	5.5
Severe (8-9)	4	2.0
Extremely severe (≥10)	3	1.5
**DASS 21 score (Stress)**		
Mild (8-9)	4	2.0
Moderate (10-12)	5	2.5
Severe (13-16)	12	6.0
Extremely severe (≥17)	1	0.5

## Discussion

Studies have demonstrated that psychiatric disorder among residents is a common phenomenon all over the world. As physician’s role is important in having a healthy society, their health and well-being is of the foremost importance. The current study was conducted to assess the extent of depression, anxiety, and stress among the residents of BSMMU, which might have policy implication. The study revealed the point prevalence of the three groups of disorders to be 15%. Among the residents, 11.5% had depressive disorders, 11% anxiety disorders, and 10.5% stress disorders (adjustment disorder and posttraumatic stress disorder). Also, cumulatively, about 15% of the residents had at least one of the three disorders. Severity distribution of the disorders revealed that 6% had severe to extremely severe depression, 3.5% severe to extremely severe anxiety, and 6.5% severe to extremely severe stress disorder. A study among Pakistani postgraduate medical trainees revealed that about 60% had depression and about 34% were moderate to markedly depressed, which is quite higher than the results of the present study (12). This variation can be explained by different study set up, different instrument, and different culture. A study in India among four medical colleges and associated hospitals of Delhi found the overall prevalence of stress to be 32.8% in resident doctors (13). Of them, 17.7% had mild stress, 12.2% moderate stress, and 2.9% severe stress. However, a study in Bangladesh found the prevalence of depression to be about 40% among postgraduate medical students among three postgraduate medical teaching institutes in Dhaka (14). Increased age, low income, marital status, living away from family, smoking, long working hours, and inadequate time for study appeared as important risk factors. A study from Turkey revealed a nearly similar depression rate to that of the present study among 156 resident physicians, where rate of probable depression was 16% (15). The rate of depression was significantly more than 5-fold higher among women compared to men and a negative correlation was also observed between depression and job satisfaction scores (15). In Malaysia, the prevalence of distressed postgraduate students was 36.4% (16). The top 10 stressors were tests and examinations, large amount of content to be learnt, time pressure to meet deadlines, doing work beyond ability, work overload, unfair assessment by the supervisor, fears of making mistakes that can lead to serious consequences, doing work that is mentally straining, work demands affecting personal and family life, and lack of time to review what have been learnt. This study found that major stressors were related to academic and performance pressure (16). Studies conducted in other different countries demonstrated that depression is an important health problem among physicians and medical faculty students (17-21). Depression among physicians is especially striking with a prevalence that rises up to 30% in the first year following graduation from the medical school (17). The high rate of psychosocial problems and depression among doctors is related to numerous factors. In the accumulation of such problem, factors related to educational and professional life stand out, as do personal characteristics (17, 22-24). A variety of reasons, which include prolonged working hours, sleep deprivation, insufficient social support, taking the responsibility of patients, financial constraint, and negative professional relationships result in such problems as excess alcohol consumption or depression among doctors (17, 22). In addition, the general assumption among doctors and medical faculty students is that they do not need the help others and that they can manage their own problems, and thus they do not use health care services when they need them (21, 23, 24). It was determined that only a very small proportion of doctors with depression receive medical treatment and instead use self-prescribed medications (17). Depression in doctors negatively affects the quality of the service they provide and causes problems in their work environment (17). In addition, psychiatric problems have negative impacts on learning and academic success in a profession where postgraduate education is highly important (25). 

## Limitation

To our knowledge, this was the first study to determine depression, anxiety, and stress related disorders among the postgraduate medical studenrs of Bangladesh. However, the present study was conducted in a single center, and thus the findings of this study cannot be generalized to reference population. The sample size was small compared to the calculated size, which does not allow generalization of the findings. Some residents did not agree to participate in the study even though they were informed about the study procedure, objectives, anonymity of data, and their right to withdraw from the study at any time, particularly those residents who had imminent exams were reluctant to participate in the interview done for data collection. This might have underestimated the prevalence of psychiatric disorders, as impending examination usually precipitates depression, anxiety, and stress among vulnerable persons. 

## Conclusion

This study revealed that one in every seven residents has been suffering from one of the three disorders (depression, anxiety, and stress related disorders). A postgraduate student counseling center may be useful to residents to help them to cope with depression, anxiety, and stress related disorders. A large-scale multicenter study is recommended to validate the findings of the present study.
